# Long-term evaluation of the implementation of a large fall and fracture prevention program in long-term care facilities

**DOI:** 10.1186/s12877-018-0924-y

**Published:** 2018-10-01

**Authors:** Patrick Roigk, Clemens Becker, Claudia Schulz, Hans-Helmut König, Kilian Rapp

**Affiliations:** 1 Department of Clinical Gerontology, Robert-Bosch-Hospital, Auerbachstrasse 110, 70376 Stuttgart, Germany; 2 Department of Health Economics and Health Services Research, University Medical Center Hamburg-Eppendorf, Hamburg, Germany

**Keywords:** Long-term care facilities, Multifactorial fall prevention program, Long-term evaluation, Implementation stage

## Abstract

**Background:**

Falls and fractures are extremely frequent in long-term care facilities (LTCFs). Therefore, a fall and fracture prevention program was started in nearly 1000 LTCFs in Bavaria/Germany between 2007 and 2010. The components of the program were exercise classes, the documentation of falls, environmental adaptations, medication reviews, the recommendation to use hip protectors and education of staff. The present study aimed to provide a comprehensive evaluation of the implementation process of the program regarding results of the implementation phase and the follow-up of 3–9 years after start of implementation.

**Methods:**

Data from numerous sources were used, including data from published studies, statistical data, health insurance claims data and unpublished data from an online questionnaire. To incorporate different aspects, time periods and results, the RE-AIM framework was applied.

**Results:**

The program was adopted by 942 of the 1150 eligible LTCFs and reached about 62,000 residents. During the implementation phase exercise classes and recommendation about environmental adaptations were offered in nearly all LTCFs. 13.5% of the residents participated in exercise classes. Hip protectors were available for 9.2% of all residents. In the first implementation wave, femoral fracture rate was significantly reduced by 18% in the first year. At follow-up nearly 90% of all LTCFs still offered exercise classes, which were attended by about 11% of residents. However, only 10% of the exercise classes completely fulfilled the requirements of an effective strength and balance training. Individual advice about environmental adaptations was provided in 74.3% of the LTCFs and nearly all LTCFs claimed to offer hip protectors to their residents. A long-term effect of the program on femoral fractures could not be detected.

**Conclusions:**

The program did not affect the femoral fracture rate in the long run. Possible reasons could be a high turn-over of the staff, a reduced fidelity of training components or a shift in daily priorities among the staff.

## Background

Long-term care facilities (LTCFs) are settings with a particularly high risk for falls. In this setting, the fall rate is reported to be about 2 falls per resident-year [1], which is considerably higher than the fall rate observed in older people living in the community [2]. In LTCFs with 90 beds, for example, a fall can be expected about every other day [3]. Therefore, fall-related injuries such as bruises, lacerations or fractures are common. One of the most serious complications of falls are femoral fractures [4]. They are particularly frequent in residents of LTCFs. In Germany, more than 20% of hip fractures are caused by residents from LTCFs even though their corresponding person-years under observation account only for 4% [5]. Therefore, there is a high interest in measures and programs which reduce the risk of falls and fall-related injuries in residents of LTCFs. At the end of the 1990s two similar cluster-randomized controlled trials from Sweden and Germany demonstrated that a multifactorial approach is able to reduce the fall rate in residents of LTCFs [6, 7]. Motivated by the results of the German trial, a large statutory health insurance company [Allgemeine Ortskrankenkasse Bayern (AOK)] decided to finance the implementation and dissemination of the program in a large number of LTCFs in Bavaria, a federal state with 12.5 million inhabitants in the south of Germany. Compared to the original study, the program components and the implementation plan of the so-called Bavarian fall and fracture prevention program (BF2P2) were somewhat modified and simplified. In total nearly 1000 LTCFs started with the BF2P2 after having signed a contract to participate in the program for at least 3 years [8].

BF2P2 was embedded in daily routine and implemented in a complex setting. To evaluate the public health significance of such an intervention a comprehensive approach which uses different methods and addresses different dimensions at different time periods is needed [9, 10]. Several analyses were published during the implementation phase of the program. They presented results on the process and on the outcome level. In addition, first results of the long-term evaluation were published recently [11]. Each of these analyses addressed only single aspects of the complex intervention. The aim of the present study is to give a comprehensive and holistic overview of the program during the implementation phase and 3–9 years after the start of the implementation by summarizing results from previous studies and by adding new results from the long-term evaluation. The Reach, Effectiveness, Adoption, Implementation and Maintenance (RE-AIM) framework was used to present the various aspects and results in a structured way [12].

## Methods

### Intervention program

The multifactorial BF2P2 aimed to reduce the fall and fracture risk in residents of LTCFs. The components of the program were progressive exercise classes of strength and balance training with weight cuffs, documentation of falls, environmental adaptations, medication reviews and prescription of Vitamin D, recommendation to use hip protectors, education of staff and educational material. Furthermore, a website was provided to present information about fall prevention and a newsletter was sent regularly to the participating LTCFs during the implementation phase [8]. Further description of the components is presented in Table 1. The program components were offered to the residents depending on their individual fall risk and physical and psychological resources.

**Table 1:** Components and details of the BF2P2

Components	Details
Exercise classes	Progressive strength training: with dumbbell at 5 different muscles of the upper part of the body and with weight-cuffs at 5 different muscles of the lower part of the body; Progressive balance training: exercises in standing position, gait variations, exercises with aids like balloons, towel or strings; 1 h twice a week; groups of 8–10 participants; to qualify for exercise groups, residents had to be able to stand with support; exercises were adapted to each resident’s capabilities; exercise instructors for the first 6 months were physiotherapists or sport therapists, supported by a member of the nursing home staff; after 6 months the training was taken over by members of the nursing home staff.
Documentation of falls	Compulsory; documentation sheets were sent to the health care insurance (AOK)^a^; regular feedback on fall statistics.
Environmental adaptations	Nurses were encouraged to look for person-environment mismatches using an environmental check list which included more than 100 items [41]
Medication review, vitamin D	Nurses were encouraged to discuss a regular medication review with the physicians focusing on reduction of inappropriate psychotropic drugs and on the prescription of vitamin D.
Hip protectors	Each home received a test kit of 5 hip protectors for demonstration purposes; recommendation of hip protectors was part of the program but they were not reimbursed by most German health care insurance companies.
Education and education materials	Change agents received a one-day training course; exercise instructors received a different one-day training course; manual with all contents of the program [13]; material for in-house education; web page with additional information [42].

^a^*AOK* Allgemeine Ortskrankenkasse Bayern (health insurance company)

To implement the BF2P2, change agents and exercise instructors were educated and trained in a one-day session [8]. The health insurance company funded an exercise instructor for each participating LTCF for 6 months to establish the training and to enable the ‘co-trainers’ (mostly nurses of the care facility) to proceed independently with the strength and balance training after the funded period. The exercise training was carried out according to a manual [13]. Additionally, care representatives of the homes (so-called change agents) served as multipliers in the LTCFs. They were supposed to take responsibility regarding fall prevention in the LTCFs, organize further training and spread their knowledge to the staff of the LTCFs. The participation in the program was voluntary for LTCFs with 35 or more beds and free for all residents irrespective of their health insurance. Each participating LTCF had to sign a contract to ensure the uptake and to implement the components of the program for at least 3 years [8].

The implementation of BF2P2 was coordinated by a statutory health insurance company, the AOK, which covers about 40% of all residents living in LTCFs in Bavaria. The program was implemented successively in four annually time-lagged implementation waves, starting in 2007, 2008, 2009 and 2010, respectively.

### Data source and data analysis

To comprehensively evaluate BF2P2, data from published analyses, data from the federal statistical office, and new and so far not yet published data were used. Data sources are briefly described below for each of the RE-AIM dimensions and also presented in Tables 3 and 4. For a more detailed description we refer to the original publications.

The evaluation of the implementation phase used data from the first and second implementation wave (2007 and 2008). Routine data of the years 2005 through 2013 were used for analysis of the long-term effectiveness of BF2P2 on femoral fractures. Additional follow-up evaluations collected data in 2015 (online-questionnaire) and 2016 (observation of exercise classes). Since the LTCFs started with the program in four annually time-lagged implementation waves and the follow-up evaluations took place at different calendar years, the follow-up period differed between different scientific questions and different LTCFs and ranged from 3 to 9 years.

### RE-AIM evaluation model

To present the different aspects and results from the start of the BF2P2 until its long-term evaluation in a structured way, the RE-AIM framework was applied [12]. This framework is used in order to understand the strengths and weaknesses of the implemented intervention and is appropriate for setting-based and public health interventions. RE-AIM is an acronym for reach (of target population), effectiveness (impact on key outcomes), adoption (among staff and settings), implementation (consistency of the intervention) and maintenance (long-term impact on individual and setting levels). The original RE-AIM definitions and the transfer of these definitions to the BF2P2 (study definitions) are explained in the following sections and presented in Table 2.

**Table 2:** Original and for BF2P2 applied study definitions of RE-AIM dimensions; variables and measurement

Dimension	Original definition	BF2P2
		Study definition
Adoption	The absolute number, proportion, and representativeness of settings and intervention agents who are willing to initiate a program.	The proportion of LTCs which participated in BF2P2
Reach	The absolute number, proportion, and representativeness of individuals who are willing to participate in a given initiative	The proportion of residents who benefited from the BF2P2
Implementation	At the setting level, implementation refers to the intervention agents’ fidelity to the various elements of an intervention’s protocol. This includes consistency of delivery as intended and the time and cost of the intervention.	Availability of components of the BF2P2 in the participating LTCFs during the implementation phase (fall and fracture prevention classes; hip protectors; environmental adaptations; medication)
Effectiveness	The impact of an intervention on important outcomes, including potential negative effects, quality of life, and economic outcomes.	Effectiveness and cost-effectiveness of BF2P2 on incident femoral fractures in the first group of LTCFs (implementation wave 1) during the first implementation year (2007)
Maintenance	The extent to which a program or policy becomes institutionalized or part of the routine organizational practices and policies. At the individual level, maintenance has been defined as the long-term effects of a program on outcomes after 6 or more months after the most recent intervention contact.	Availability and quality of components of the BF2P2 in the participating LTCFs during follow-up (fall and fracture prevention classes; hip protectors; environmental adaptations; medication); Long-term effect of BF2P2 on incident femoral fractures in all implementation waves between 2007 and 2013

### Adoption

The dimension ‘Adoption’ describes the proportion of LTCFs which participated in BF2P2 and was calculated by dividing the number of participating LTCFs by the number of eligible LTCFs with 35 or more beds. The number of participating LTCFs was obtained from the health insurance company AOK, the number of eligible LTCFs from the federal statistical office [14].

### Reach

The dimension ‘Reach’ describes the proportion of residents who benefited from the BF2P2. Since BF2P2 aimed to influence the whole setting of the LTCF by its multifactorial approach, all residents of the participating LTCFs were supposed to benefit in one way or another. Therefore, reach was calculated by dividing the number of residents of participating LTCFs by the number of residents of eligible LTCFs with 35 or more beds. The number of residents was estimated using data from the federal statistical office [15].

### Implementation

In our study the dimension ‘Implementation’ is operationalized by the availability of the following components of the program in the participating LTCFs during the implementation phase: Fall and fracture prevention classes, hip protectors, environmental adaptations and medication. Furthermore, costs of the BF2P2 were assessed.

In 2008, telephone interviews were conducted with change agents (in most cases care managers) from 69 randomly selected participating LTCFs. They were asked about the availability of fall and fracture prevention classes, the acquisition of hip protectors, and if recommendations about environmental adaptations were routinely offered to their residents. This and additional information was used to calculate the incremental costs of the program during the first 18 months of the implementation [16]. At the same time, a nursing scientist visited 48 randomly selected participating LTCFs for 1 day and collected information from 4000 residents about each resident’s participation in exercise classes and about each resident’s availability and use of hip protectors [17]. The information referred to the previous 4 weeks and was provided by the nursing home staff for each resident. Implemented components are presented as percentages.

### Effectiveness

The dimension Effectiveness was determined by analyzing the effect of the BF2P2 on incident femoral fractures (ICD-10: S72) during the first intervention year (2007; implementation wave 1). Femoral fracture rates were compared between 13,653 residents from 256 LTCFs which started with BF2P2 during wave 1 (intervention-LTCFs), and 31,668 residents from 893 remaining LTCFs which started during later waves (control-LTCFs). Since LTCFs were not randomized, the selection of LTCFs may have influenced the outcome. Therefore, femoral fracture rates were also calculated for the years before the start of the intervention (2001, 2002, 2003, 2004, 2005, and 2006) and compared between intervention-LTCFs and control-LTCFs. The femoral fracture-related costs and intervention costs were measured from a payer perspective [18]. Claims data provided by AOK served as data source to analyze Effectiveness and Cost-effectiveness.

### Maintenance

The dimension ‘Maintenance’ is defined as the availability and quality of components of the BF2P2 in the participating LTCFs during follow-up and the Long-term effect of BF2P2 on incident femoral fractures in all implementation waves. The dimension was evaluated during follow-up in three different ways: First, by assessing the availability of fall prevention measures in the long run, second, by analyzing the fidelity of exercise components in the fall and fracture prevention classes according to the initial protocol, and third, by evaluating the long-term effect of the program on incident femoral fractures (ICD-10: S72).

The availability of fall prevention measures was assessed by an online-questionnaire which was sent to the facility managers or care managers of all participating LTCFs in October 2015. As BF2P2 was implemented time-lagged, this time point was 3 to 9 years after implementation for wave 1 to 4, respectively. The questionnaire asked if the initially educated change agents and exercise instructors were still working in the LTCF, if fall and fracture prevention classes were currently available, if hip protectors were currently made available by the LTCFs, if individual advice about environmental adaptations was routinely provided, and if nurses still discussed the residents’ medication with the general practitioner (GPs). The response rate to the online questionnaire was 17.7% (*N* = 167). Data from the online-questionnaire have not been published so far.

To evaluate the fidelity of exercise components according to the “Ulmer Modell” a sport scientist visited 40 different classes in 40 randomly selected LTCFs. Each class was visited once between January 2016 and June 2016. The observation of each training session was recorded in a standardized observation sheet and included type, quality and frequency of specific exercise components [19].

To analyze the effect of BF2P2 on incident femoral fractures (ICD-10: S72) from 2005 through 2013, health insurance claims data of 85,148 residents from 802 nursing homes were used. LTCFs of all four implementation waves were incorporated in a comprehensive unbalanced panel data set. For each of the implementation waves, 2 years prior to implementation of BF2P2 were used as baseline and the following 4 years were investigated. The likelihood of a femoral fracture was estimated for every intervention year relatively to the baseline years before BF2P2 started. Fracture rates were standardized to sex, age, and the degree of dependence (care level) [11].

## Results

### Adoption

Of all 1633 Bavarian LTCFs, 1150 LTCFs met the inclusion criterion of having at least 35 beds. Between 2007 and 2010, 942 of the 1150 eligible LTCFs implemented the LTCFs program, which corresponds to a participation rate of more than 80% (Table 3).

**Table 3:** Dimensions Adoption, Reach, Implementation and Effectiveness of the RE-AIM framework of the BF2P2 during the implementation period (2007–2010)

RE-AIM dimension	Parameters of the RE-AIM dimension	Data source and time period
Adoption		
All LTCFs in Bavaria, N	1633	• Statistic data from the federal statistical office [14]
Eligible LTCFs (≥35 beds) among all LTCFs in Bavaria, n (%)	1150 (70.4)	
Participating LTCFs (Adopters) per eligible LTCFs, n (%)	942 (81.9)	• Claims data by AOK^a^
Reach		
Residents living in all LTCFs in Bavaria, N	107,507	• Statistic data from the federal statistical office [15]
Residents living in eligible LTCFs (≥35 beds), n (%)	75,685 (70.4)	
Residents of participating LTCFs, n (%)	61,986 (81.9)	
Implementation		
Fall and fracture prevention classes		
Interviewed facility- or care manager of participated LTCFs, N	69	• Telephone interviews in 69 LTCFs in 2008 [16]
LTCFs offering classes, n (%)	67 (97.6)	
Residents in observed LTCFs, N	4000	• Field visits in 48 LTCFs in 2008 [8]
Residents attending the classes, n (%)	540 (13.5)	
Hip protectors		
Residents in observed LTCFs, N	3924	• Field visits in 48 LTCFs in 2008 [17]
Residents owning hip protectors, n (%)	361 (9.2)	
Use of hip protector during the last 4 weeks if available, n (%)	229 (63.6)	
Environmental adaptations		
Interviewed facility- or care manager of participated LTCFs, N	69	• Telephone interviews in 69 LTCFs in 2008 [16]
LTCFs in which individual advice about environmental adaptations was provided regularly, n (%)	68 (98.6)	
Costs		
Additional costs caused by the implementation of the program within the first 18 month, € (SD)	6248 (±7340)	• Telephone interviews in 69 LTCFs in 2008 [16]
Effectiveness		
Risk of femoral fractures (intervention LTCFs vs. control LTCFs) in the first implementation wave in 2007, HR (95%CI)	0.82 (0.72–0.93)	• Claims data by AOK^a^ from 256 LTCFs / 13,653 residents in 2007 [8]
Incremental cost-effectiveness ratio (ICER), € per year free of femoral fracture	7.481	• Claims data by AOK ^A^ from 256 LTCFs/ 10,178 residents in 2007 [18]

*n* number of residents who participated in fall and fracture prevention classes and used hip protectors and number of participated LTCs who offered the program components of the BF2P2, *LTCFs* Long term care facilities (in Bavaria)

^a^*AOK* Allgemeine Ortskrankenkasse Bayern (health insurance company)

### Reach

Since no residents of participating LTCFs were excluded from BF2P2, the program reached about 62,000 (81.9%) residents out of more than 75,000 eligible residents in Bavaria (Table 3).

### Implementation

One of the core components of the program were exercise classes for strength and balance training. Nearly all of the participating LTCFs offered such classes (97.6%). During the implementation period 13.5% of the residents (range 3.4 to 47.8% per LTCF) participated in exercise classes.

Hip protectors were made available for 9.2% of all residents. The availability of hip protectors varied considerably between LTCFs. In 25% of the LTCFs hip protectors were not present at all, whilst in other LTCFs more than 25% of the residents owned hip protectors. However, only 63% of the residents who owned hip protectors actually used them in the 4 weeks prior to the data collection.

Recommendations about environmental adaptations were routinely offered in nearly all LTCFs.

The implementation of the complete program caused additional costs of 6248 EUR (± SD 7340 EUR; pricing year 2008) per LTCF within the first 18 months (Results are displayed in Table 3).

### Effectiveness

During the first intervention year 2007, the femoral fracture rate was significantly reduced by 18% in the first implementation wave compared to the remaining group of LTCFs not yet participating in the program (Table 3).

The incremental cost-effectiveness ratio which was calculated by the difference in mean costs and mean effects at the group level (ICER) was 7481 EUR per year free of femoral fracture. The net benefit turns into a positive value if the willingness-to-pay (WTP) amount reaches around 7500 EUR, which reflects the point estimate of the adjusted cost-effectiveness ratio (ICER) (Table 3).

### Maintenance

During follow-up less than 20% of the initially educated change agents, who were supposed to serve as multipliers in the facilities, were still available. 55.5% of the current exercise instructors had received a specific education regarding the contents of the exercise program during the implementation phase (Table 4).

**Table 4:** Maintenance of the BF2P2 fall prevention components, fidelity of the training components and long term results in fracture incidence in participating LTCFs during follow-up

RE-AIM dimension	Parameters of the RE-AIM dimension	Data source and time period
Maintenance of the implemented components		
LTCFs in which the primarily educated personnel was still available		
Interviewed facility- or care manager of participated LTCFs, N	110	Online questionnaire in 167 LTCFs in 2015
Change agent of the LTCFs, n (%)	21 (19.1)	
Exercise instructor of the LTCFs, n (%)	61 (55.5)	
Fall and fracture prevention classes		
Interviewed facility- or care manager of participated LTCFs, N	167	Online questionnaire in 167 LTCFs in 2015
LTCFs offering classes, n (%)	146 (87.4)	
Residents in observed LTCFs, n	4013	Structured observation in 40 LTCFs in 2016 [19]
Residents attending the classes, n (%)	432 (10.8)	
Fidelity of the training components		
Observed LTCFs, N	40	Structured observation in 40 LTCFs in 2016 [19]
Classes which used weight cuffs, n (%)	12 (30.0)	
Classes with an appropriate number (≥6) of balance exercises, n (%)	10 (25.0)	
Classes which completely fulfilled the requirements of an effective strength and balance training, n (%)	4 (10)	
Hip protectors		
Interviewed facility- or care manager of participated LTCFs, N	167	Online questionnaire in 167 LTCFs in 2015
Number of LTCFs offering hip protectors to their residents, n (%)	156 (93.4)	
Residents in interviewed LTCFs, n	15,577	
Numbers of residents’ for whom hip protectors were made available by the LTCFs, n (%)	2950 (18.9)	
Environmental adaptations		
Interviewed facility- or care manager of participated LTCFs, N	167	Online questionnaire in 167 LTCFs in 2015
LTCFs in which individual advice about environmental adaptations was provided regularly, n (%)	124 (74.3)	
Medication		
Interviewed facility- or care manager of participated LTCFs, N/N	165/146	Online questionnaire in 167 LTCFs in 2015
Staff talked to GPs about residents’ medication ‘frequently’^a^, n (%)	117 (70.9)	
Staff talked to GPs about Vitamin D prescriptions ‘frequently’^a^, n (%)	19 (13.0)	
Long-term effectiveness of fracture prevention		
Intervention year,		
Implementation wave 1		
Two years before the intervention (baseline), OR	1.00	Claims data by the AOK^b^ from 802 LTCFs/ 85,148 residents from 2005 to 2013 [11]
First year of BF2P2, OR (95%CI)	0,87 (0.75–0.99)	
Second year of BF2P2, OR (95%CI)	0,80 (0.62–1.04)	
Third year of BF2P2, OR (95%CI)	0,92 (0.82–1.02)	
Fourth year of BF2P2, OR (95%CI)	0,99 (0.84–1.18)	
Implementation waves 1–4, combined		
Two years before the intervention (baseline), OR	1.00	
First year of BF2P2, OR (95%CI)	0.96 (0.89–1.05)	
Second year of BF2P2, OR (95%CI)	0.96 (0.84–1.10)	
Third year of BF2P2, OR (95%CI)	0.97 (0.91–1.03)	
Fourth year of BF2P2, OR (95%CI)	1.04 (0.95–1.15)	

*n* number of residents who participated in fall and fracture prevention classes and used hip protectors and number of participating LTC’s which offered the program components of the BF2P2, *LTCFs* Long term care facilities (in Bavaria)

*OR (95%CI)* Odds ratio (95% confidence interval)

^a^‘frequently’ combines the two answer categories ‘always’ and ‘often’ of a 5-point Likert scale (always; often; sometimes; seldom; never)

^b^*AOK* Allgemeine Ortskrankenkasse Bayern (health insurance company)

Nearly 90% of the LTCFs still offered exercise classes and about 11% of the residents from all participating LTCFs which offered exercise classes attended exercise classes at the follow-up assessment. Most of the exercise instructors (62.5%) had a qualification in nursing (registered nurse or nursing assistance), a smaller proportion (37.5%) in physiotherapy or occupational therapy. Components of strength training were a frequent part of the training (Table 1). However, only 30% of the classes used the recommended weight cuffs for the strength training of the lower extremities. Furthermore, balance exercises were only sparsely or not at all performed in many of the classes. The balance exercises were performed more frequently when exercise instructors had a therapeutic qualification. Only 10% of the exercise classes completely fulfilled the requirements of the fall prevention training according to the given standards of the ‘Ulmer Modell’ (Table 4).

At the follow-up assessment nearly all LTCFs claimed to offer hip protectors to their residents. The percentage of residents for whom hip protectors were made available by the LTCFs was nearly 20% (Table 4). This is clearly higher than the availability during the implementation phase. However, we had no information how many residents actually used hip protectors.

Individual advice about environmental adaptations was provided in 74.3% of the LTCFs (Table 4).

In more than two thirds of the LTCFs (70.9%) nurses discussed the residents’ medication frequently with the GP. However, the prescription of Vitamin D was part of the discussion within the ward round only in a few LTCFs (Table 4).

As described above, the femoral fracture rate was significantly reduced in the first intervention year of the first wave. For the same wave, a reduction was also observed in the second and partly in the third year. In the fourth year, which was the first year after the funded implementation phase ended, a reduction of femoral fractures was no longer observable. In contrast, the intervention was not associated with a significant reduction of femoral fractures in any year of the waves 2, 3 and 4. Therefore, only a transient reduction of femoral fractures in only the first implementation wave was observed whilst a long-term effect of BF2P2 in terms of reducing femoral fractures could not be detected (Table 4).

## Discussion

This paper provides a comprehensive evaluation of the fall and fracture prevention program BF2P2. The majority of all Bavarian LTCFs adopted the program and the majority of residents were reached. Numerous core components of BF2P2 like recommendations about environmental adaptations or exercise classes were implemented. We found a transient reduction of femoral fractures in the first implementation wave, but no effect on femoral fractures in the following waves. Even after a follow-up of 3–9 years, most of the intervention components were still available. However, the initially educated instructors and change agents were often not available any more due to a high turnover of LTCF staff. A long-term effectiveness of BF2P2 over all Bavarian LTCFs could not be detected.

There are only few studies which analyzed the implementation of fall prevention measures in routine care of LTCFs. They usually focused on specific aspects like effectiveness [20, 21], uptake of and adherence to exercise classes [22] or on facilitators and barriers of using hip protectors [23]. To the best of our knowledge the BF2P2 is the so far largest implementation program for fall and fracture prevention in LTCFs and also the first program with such a comprehensive evaluation including different methods, levels and time periods.

More than 80% of all eligible LTCFs in Bavaria implemented the program. The extremely high adoption rate may be attributed to the publication of a new standard for nurses in fall prevention in 2006 [24] which had to be realized in the facilities from 2009 on [§113a SGB XI]. Therefore, the education of change agents and exercise instructors and the financial support of the exercise classes over 6 months by the health insurance company were considered as an opportunity to transfer evidence based knowledge into practice in a structured way. The degree of implementation of most of the components was also high. For example, nearly all LTCFs offered exercise classes and individual environment counsels.

Therefore, it was disappointing that we observed only a transient reduction of femoral fractures in only the first implementation wave whilst a long-term reduction of femoral fractures could not be detected. Since the study was not randomized, the restriction of the transient effect of the intervention to the first implementation wave may be explainable by a higher motivation of those LTCFs starting first with the program. This suggests that the program is in principle able to reduce femoral fractures, if the motivation of the institutions and the staff is high. The transient reduction in the first wave also shows that it seems to be difficult to maintain a high standard in fall and fracture prevention over a period of several years.

Our comprehensive evaluation over several years revealed different reasons for the failure of a long-term reduction of femoral fractures. First, the components of the program affected the residents in different ways and with different intensity. During the implementation phase, for example, only 13.5% of all residents participated in training classes. One reason is that the exercise classes can be only attended if the resident’s functional and cognitive status allows at least standing with support and following the instructions of the exercise instructor. However, the participation rate was clearly lower than 25%, which was mentioned in the study by Becker et al [6]. The health insurance company financed only one exercise class per LTCF independently from its size. This may have excluded eligible residents from exercise due to capacity restrictions.

Second, quality and fidelity of the training program was often not sufficient. In addition, the progressive nature of the exercises may often not have been realized. This may be explained by the high turnover [25, 26] of exercise instructors, which means a loss of expertise. Also, the observed heterogeneity of residents’ functional and cognitive status in LTCFs [27] may be a reason. This would force exercise instructors to adapt their training to the needs and abilities of participants, who may be more functionally impaired than presumed.

Third, the program recommended hip protectors in high-risk residents but provided only a test-kit of five hip protectors for each LTC. Since hip protectors are not reimbursed by health insurance companies, only a minority of residents owned hip protectors and even a lower percentage (5.8%) used them regularly during the implementation phase. At the time of the follow-up evaluation, most of the LTCFs were able to offer their residents hip protectors from an own pool. This underlines that fall and fracture prevention is still on the facilities’ agenda. Unfortunately, the currently available hip protectors in the LTCFs of our study did not have a significant effect on the femoral fracture rate. This contributes to the discussion about the effectiveness of hip protectors in daily routine of long-term care [28, 29]. Possible reasons for a lack of effectiveness of hip protectors in LTCFs may be poor adherence by the residents, differing attitudes of the staff regarding their benefit, and different brands with different biomechanical properties.

Fourth, discussion about the appropriateness of drugs with the GPs was stated to be performed in 70% of the LTCFs at follow-up but we question that it was actually done in 70% of the individuals. On the one hand nurses may feel uncomfortable to discuss a resident’s medication with GPs [30, 31], on the other hand the prescription or termination of fall-inducing drugs like neuroleptics or benzodiazepines are often triggered by information given by nurses. Particularly rare was the discussion about the prescription of vitamin D which is known to have a beneficial effect on bone quality and on fall risk in people with low serum levels [32]. We do not know if it was discussed more frequently during the implementation phase since this component was only assessed at follow-up.

Fifth, since the publication of the new standard in fall prevention in 2006 seven additional standards dealing with other topics like nutrition- or pain management and had also to be implemented into daily routine in the LTCFs. This may have lowered priority of fall prevention in long-term care. Furthermore, the promotion of physical activity is also a priority in long-term care [33, 34]. Physical activity has many benefits and may increase autonomy and self-determination but in case of a poor quality of gait or risk taking behaviour it can also interfere with the aim of preventing falls.

The BF2P2 may have had beneficial effects like an increase of social contacts, quality of life or physical function and physical activity. These effects were not measured in the BF2P2 but well known from other fall prevention trials [35, 36]. Nevertheless, our approach failed to give a sustainable solution how the huge burden of fall-related injuries in LTCFs can be reduced on a population level. The one-day training session for the change agents and the exercise instructors may be far too little to change the culture within LTCFs, even though the change agents were supposed to act as multipliers. Therefore, different strategies and an increase of intensity, quantity and repetition of the education over a longer time period may be an approach for the future [37]. This would be associated with a considerable additional investment. Another approach could be to reconsider the measures introduced so far. New generations of LTCFs, which are smaller and differ in architecture and care-concepts from facilities of the past [38], may offer new opportunities for more effective measures. Examples could be architectural solutions for a better supervision of residents at risks, compliant flooring [39] or partnerships with other organizations (e.g. sport associations offering additional exercise classes) [40].

A strength of the current evaluation is our comprehensive approach which analyzed process and outcome variables, included different methods and time periods and used a standardized framework [12] for the reporting of the results in a structured way. It covers a highly relevant topic of the public health sector and evaluates a large program which is included in daily routine. The data are representative and cover a complete federal state.

A weakness of our evaluation is that the availability of some of the program components such as fall and fracture prevention classes or hip protectors were assessed by different instruments during implementation phase and follow-up. This limits the comparability of the results. Furthermore, only 17.7% of the LTCFs completed the online questionnaire at follow-up which could have biased our results. The conditions in LTCFs differ from country to country and from healthcare system to healthcare system. This limits the generalizability of our results.

## Conclusion

In conclusion, the BF2P2 did not affect the femoral fracture rate in the long run. However, we observed a transient reduction of femoral fractures in the first implementation wave. This suggests that the dissemination of an evidence-based program into routine care is principally able to reduce femoral fractures. We identified different reasons which may have limited the effectiveness of the program like a high turn-over of staff, a reduced fidelity of training components or a shift in daily priorities among the staff. Fracture prevention in long-term care remains a challenge. A higher investment to guarantee a sustainable change of the implemented measures and processes in LTCFs or the introduction of completely new measures could be perspectives for the future.

## Abbreviations

AOK: Allgemeine Ortskrankenkasse
BF2P2: Bavarian fall and fracture prevention program
GP: General Practitioner
ICER: Incremental cost-effectiveness ratio
LTCFs: Long-term care facilities
RE-AIM framework: Reach, Effectiveness, Adoption, Implementation and Maintenance framework
WTP: Willingness-to-pay
